# Recurrent Multifocal Urothelial Carcinoma: A Case Report

**DOI:** 10.7759/cureus.104653

**Published:** 2026-03-04

**Authors:** Pedro Serrano, Pedro Barros, Marco Dores, Aníbal Coutinho

**Affiliations:** 1 Department of Urology, Hospital de Faro, Faro, PRT

**Keywords:** bulbar urethra, multifocal disease, recurrence, suspected lynch syndrome, urothelial carcinoma

## Abstract

We report the case of a 60-year-old man with a history of prostate cancer treated with external beam radiotherapy at the age of 47, who subsequently developed recurrent multifocal urothelial carcinoma involving the bladder, ureter, and left kidney, with later recurrence in the bulbar urethra.

The patient had pre-existing chronic kidney disease prior to undergoing bilateral nephroureterectomy and subtotal cystectomy as salvage procedures for radiation-induced cystitis with refractory hematuria. He later presented with urethrorrhagia, and urethroscopy revealed papillary lesions at the bulbo-membranous junction. Perineal urethrectomy failed to control bleeding, necessitating salvage prostatectomy for definitive management.

This case highlights the clinical and surgical challenges associated with recurrent multifocal urothelial carcinoma and emphasizes the importance of a multidisciplinary approach in complex urological oncology cases.

## Introduction

Upper tract urothelial carcinoma (UTUC) is a relatively rare malignancy, accounting for approximately 5-10% of all urothelial cancers worldwide. Recent epidemiological data estimate an annual incidence of approximately two cases per 100,000 population in Western countries, with higher rates observed in selected high-risk groups. UTUC may present as unifocal or multifocal disease involving the renal pelvis and ureter, often limiting conservative management options and requiring radical surgical treatment. Multifocality is associated with an increased risk of recurrence throughout the urothelial tract, including the bladder and urethra [1-2].

Radical nephroureterectomy remains the standard of care for high-grade or invasive UTUC. However, management becomes particularly challenging in patients with a solitary functioning kidney or advanced chronic kidney disease, especially in the presence of persistent or refractory hematuria [3]. Patients with multifocal urothelial carcinoma are also at increased risk of subsequent bladder or urethral recurrence, frequently necessitating a multimodal and sequential therapeutic approach [4].

We present a complex case illustrating these challenges in a patient undergoing chronic hemodialysis, highlighting the uncommon occurrence of sequential multifocal recurrence involving the upper tract, bladder, and prostatic urethra, and the surgical decision-making required in this context.

## Case presentation

A 60-year-old obese man who was an active smoker (20 cigarettes per day), with a history of prostate adenocarcinoma treated with external beam radiotherapy in 2011 and high-grade non-muscle-invasive bladder cancer diagnosed in 2017, previously managed with multiple transurethral resections [5], was referred for refractory hematuria.

In late 2023, hematuria persisted despite repeated endoscopic management and fulguration, initially attributed to radiation cystitis versus bladder cancer recurrence. Computed tomography (CT) revealed known right renal atrophy and a 3 cm solid lesion in the left renal pelvis, highly suggestive of urothelial carcinoma. Additionally, left ureteral wall thickening with a suspected intraluminal lesion in the mid-ureter was observed, consistent with multifocal upper tract urothelial carcinoma.

**Figure 1:**
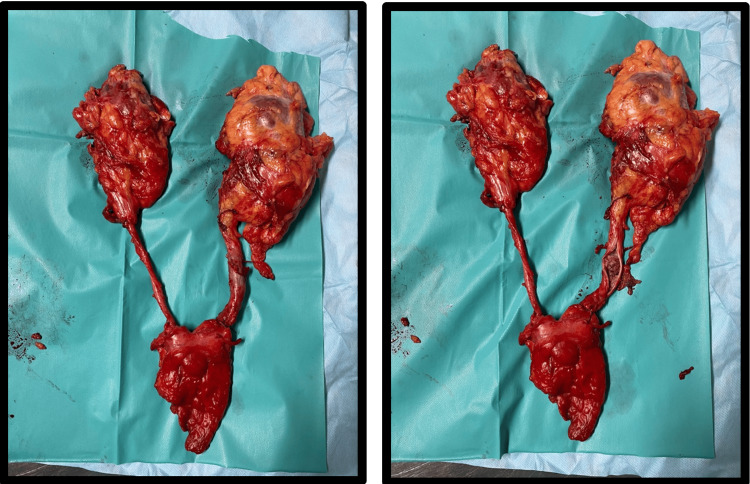
Specimen from bilateral nephroureterectomy and subtotal cystectomy performed in January 2024 for refractory hematuria and multifocal urothelial carcinoma.

Given the presence of end-stage chronic kidney disease requiring hemodialysis and the absence of metastatic disease, the patient underwent bilateral nephroureterectomy and salvage subtotal cystectomy in January 2024 (Figure 1). The right kidney was previously known to be markedly atrophic and nonfunctional. Considering the patient’s dialysis-dependent status and the need for definitive hemorrhagic and oncologic control, bilateral upper tract removal was deemed appropriate. Subtotal cystectomy was selected primarily for hemorrhagic control in the setting of radiation-induced cystitis, taking into account prior pelvic radiotherapy and technical considerations. Thus, preservation of limited bladder remnants was considered acceptable.

Histopathological examination revealed multiple foci of low- and high-grade papillary urothelial carcinoma involving the renal pelvis and ureter, without evidence of muscular invasion. Surgical margins were negative. No lymphovascular invasion was identified, and carcinoma in situ was not reported in the examined specimens.

**Figure 2:**
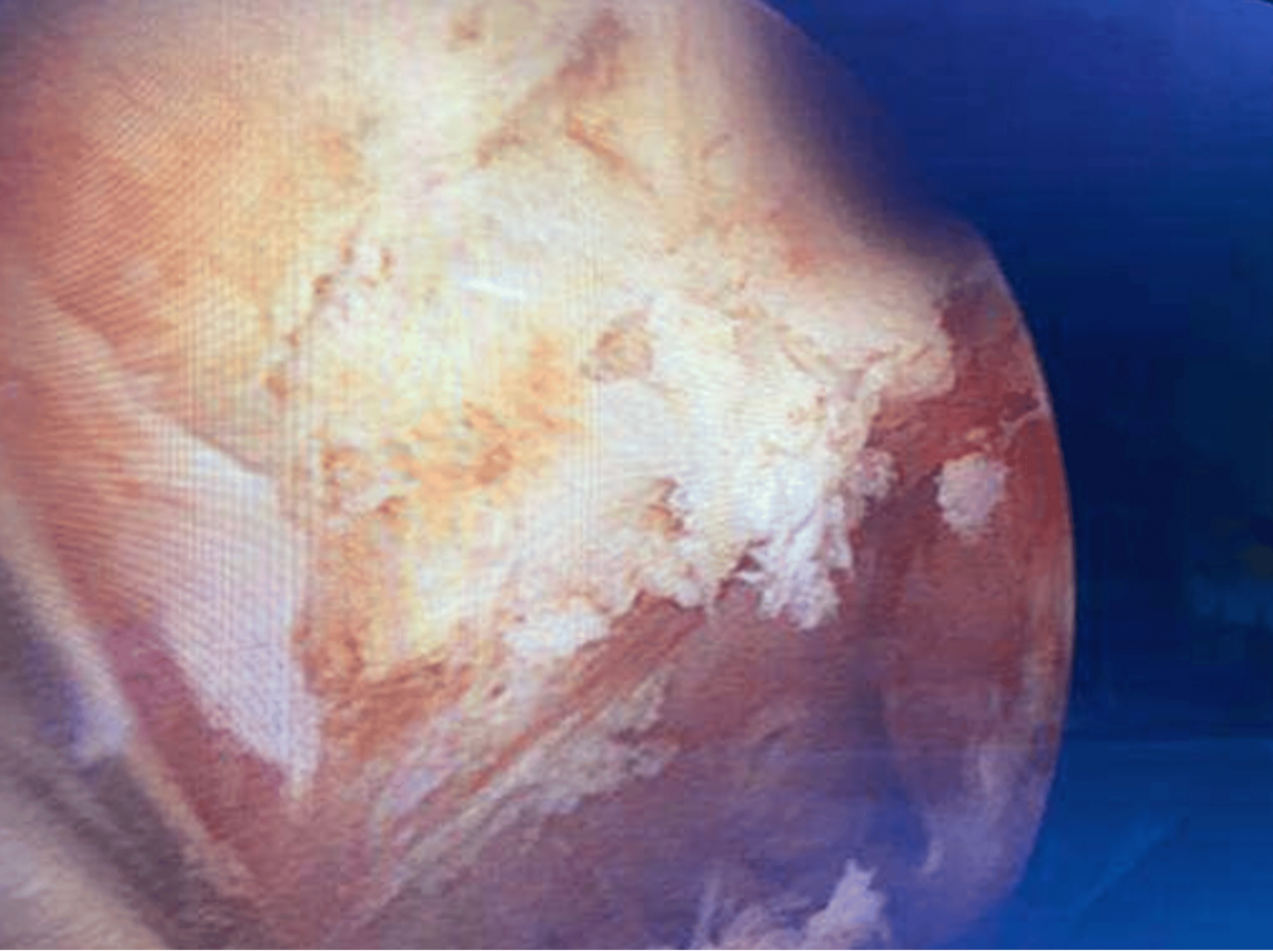
Urethroscopy performed in January 2025 demonstrating a recurrent papillary lesion at the bulbo-membranous junction of the urethra.

In January 2025, the patient presented with intermittent urethrorrhagia. Urethroscopy identified a superficial-appearing tumor at the bulbo-membranous junction (Figure 2).

**Figure 3:**
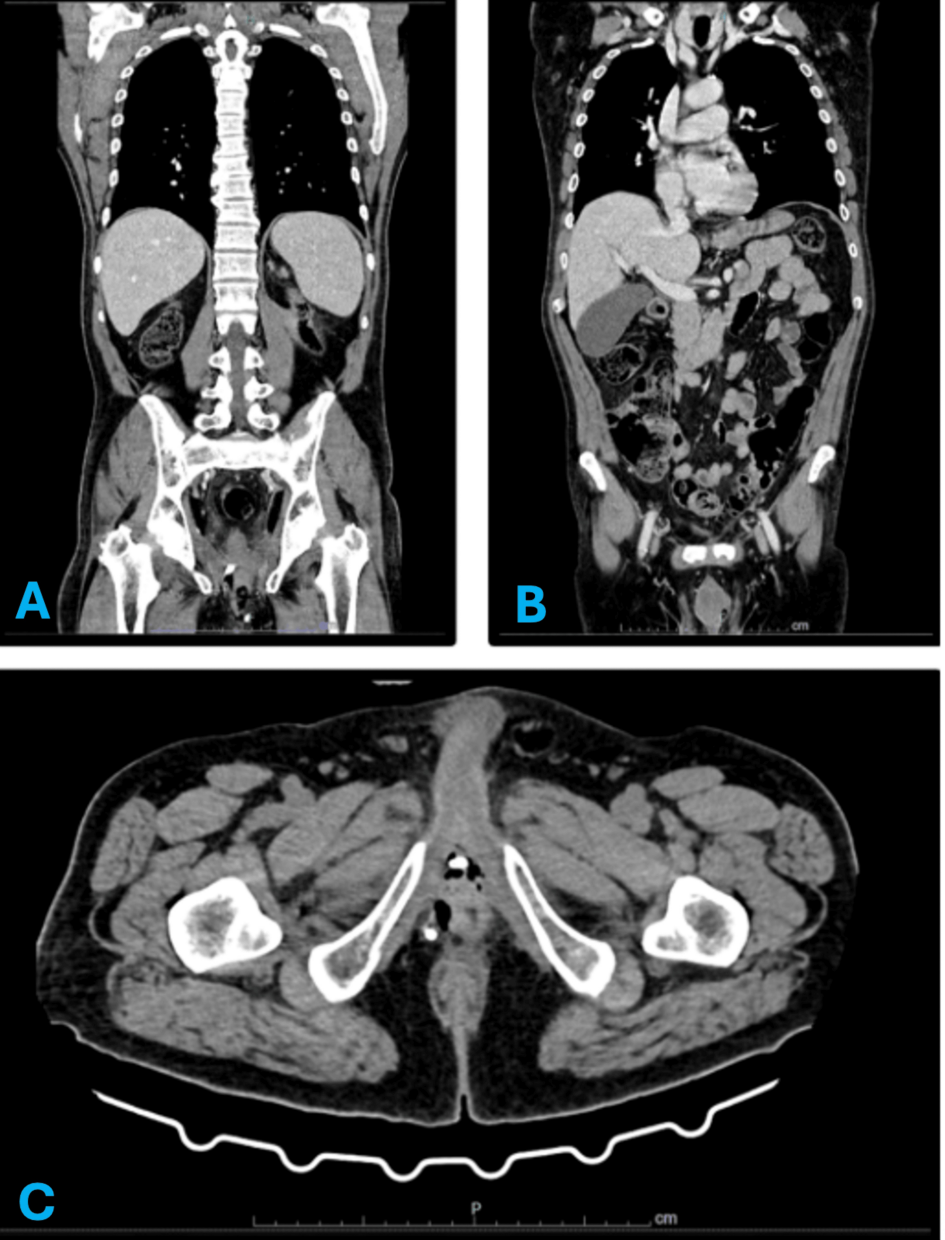
Contrast-enhanced thoraco-abdominal-pelvic CT scan performed in January 2025. (A) Coronal reconstruction demonstrating the upper abdomen without evidence of distant metastatic disease. (B) Coronal reconstruction at a different anatomical level confirming the absence of thoracic or abdominal metastases. (C) Axial pelvic section showing no evidence of local pelvic recurrence or lymph node enlargement.

Thoraco-abdominal-pelvic CT showed no evidence of distant disease (Figure 3).

**Figure 4:**
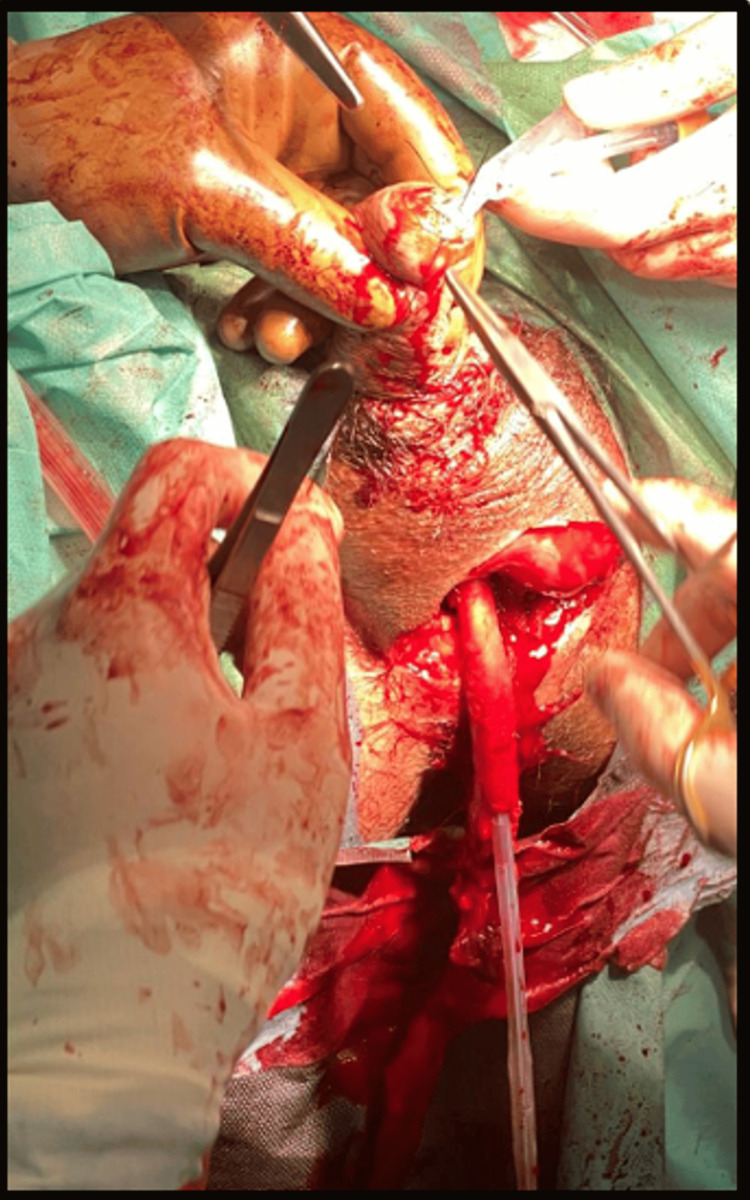
Intraoperative view of perineal urethrectomy performed in January 2025 for local disease control.

Perineal urethrectomy was performed for local disease control (Figure 4).

**Figure 5:**
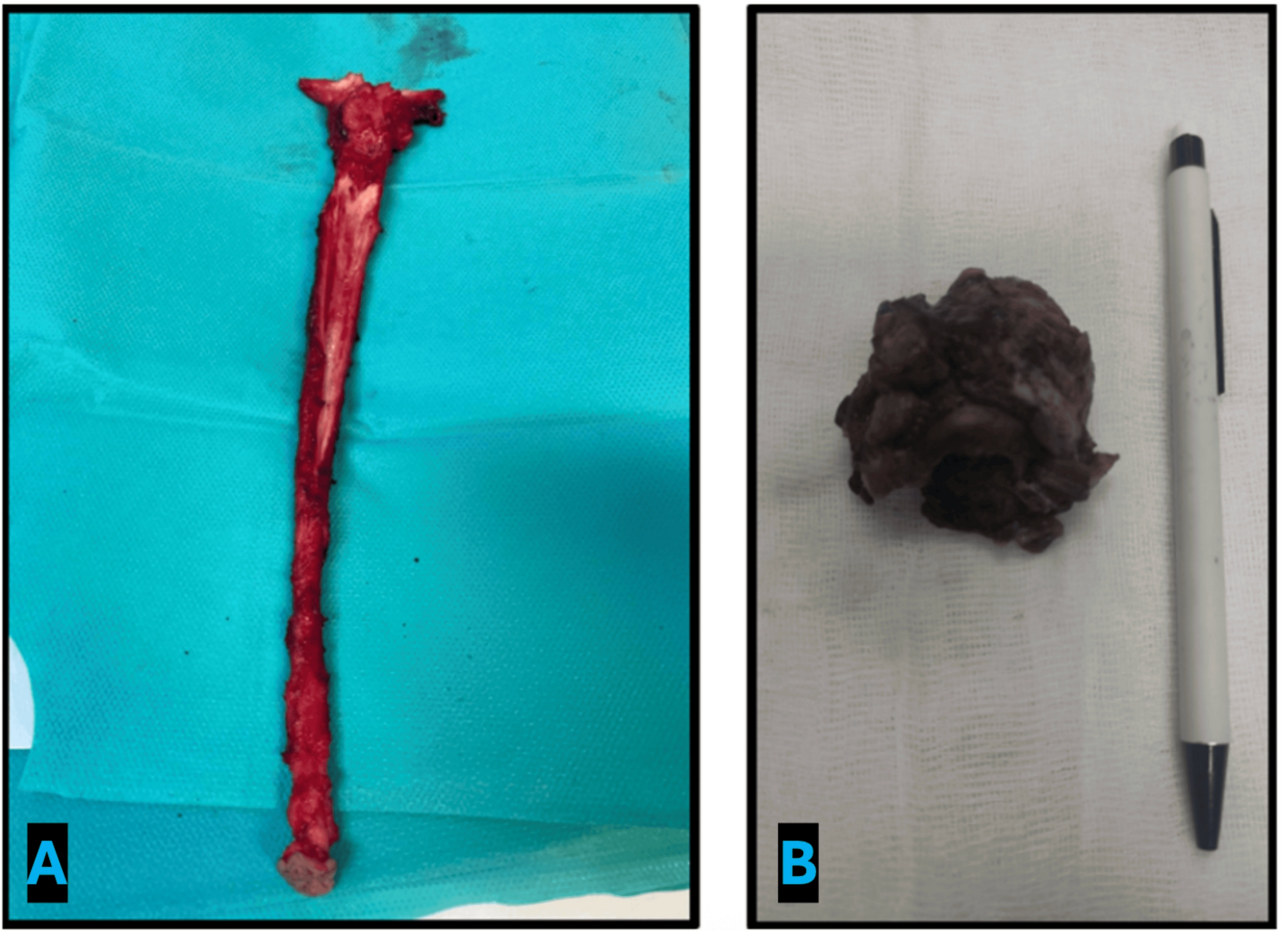
Surgical specimens obtained in 2025. (A) Specimen from perineal urethrectomy performed for local disease control. (B) Specimen from the subsequent salvage prostatectomy.

Histological analysis of the excised urethral segment revealed no residual malignancy, and programmed death-ligand 1 (PD-L1) expression assessed by immunohistochemistry was negative. Despite the absence of residual malignancy in the urethrectomy specimen, persistent perineal bleeding and suspicious endoscopic findings at the level of the prostatic urethra maintained concern for residual disease. The decision to proceed with salvage prostatectomy was made after multidisciplinary discussion, considering the ongoing hemorrhagic symptoms and oncologic risk (Figure 5).

**Figure 6:**
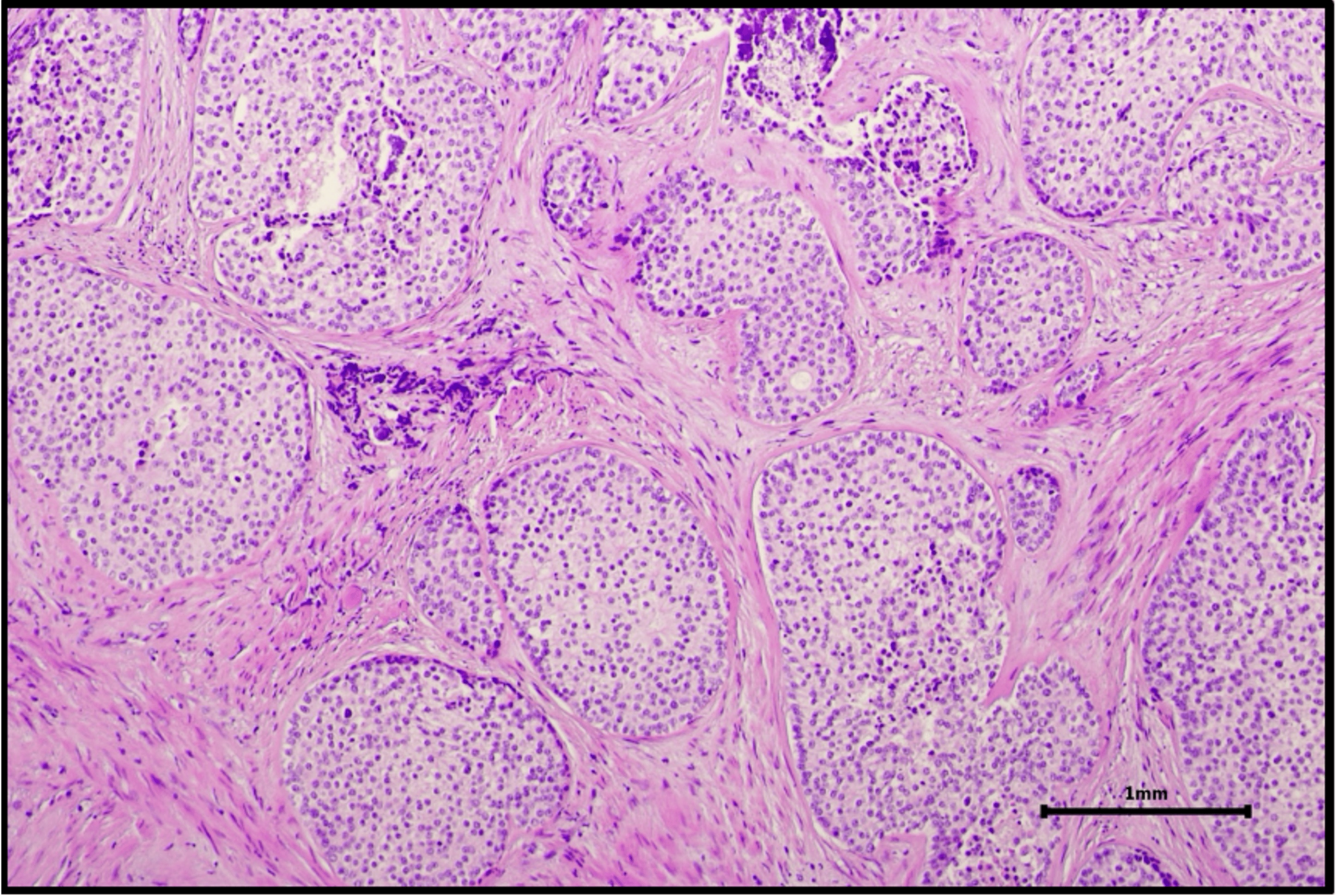
Histopathological section of the prostatic urethra demonstrating urothelial carcinoma (hematoxylin and eosin stain, original magnification ×100).

Histopathological examination of the prostatectomy specimen (Figure 6) confirmed residual prostate adenocarcinoma, previously treated with radiotherapy, and concomitant urothelial carcinoma centered in the prostatic urethra with stromal invasion consistent with pT2 staging. The tumor involved the prostatic urethral mucosa and extended into the underlying prostatic stroma.

Genetic evaluation was requested to exclude Lynch syndrome, which is associated with an increased risk of multiple malignancies, including urothelial and prostate carcinoma. Microsatellite instability testing of previously resected surgical specimens is currently ongoing.

This investigation is being conducted in the context of multifocal urinary tract neoplasia with recurrent disease at multiple anatomical sites. The patient remains under close oncological surveillance. To date, screening for other Lynch syndrome-associated malignancies, particularly colorectal carcinoma, has been negative.

## Discussion

This case illustrates the complexity of managing recurrent multifocal urothelial carcinoma, particularly in patients with advanced renal impairment, in whom clinical presentation may be atypical due to reduced urine output. In this context, bilateral nephroureterectomy combined with subtotal cystectomy represented a necessary salvage strategy to achieve both oncological and hemorrhagic control.

Urethral recurrence, although frequently superficial, reflects the field-wide vulnerability of the urothelium to malignant transformation [6]. While urethrectomy is generally considered the standard treatment, persistent bleeding and suspected involvement of the prostatic urethra justified salvage prostatectomy, enabling complete excision of the remaining urothelial tissue at risk.

Negative PD-L1 expression may limit eligibility for certain immunotherapeutic strategies. PD-L1 expression was evaluated in anticipation of potential systemic immunotherapy in the event of disease progression or metastatic recurrence. Although systemic treatment was not initiated at that stage, the result was considered clinically relevant for future therapeutic planning.

The suspicion of Lynch syndrome, prompted by early-onset prostate cancer and synchronous urothelial malignancies, underscores the importance of genetic assessment in similar clinical contexts. At present, Lynch syndrome remains unconfirmed, and microsatellite instability testing of prior surgical specimens is ongoing. This distinction between suspected and confirmed hereditary cancer predisposition has been clarified throughout the manuscript [7,8].

Beyond the surgical sequence itself, this case reinforces the concept of field cancerization within the urothelial tract. Sequential involvement of the upper tract, bladder, and prostatic urethra supports the hypothesis of pan-urothelial susceptibility rather than isolated tumor events.

Although intraluminal tumor seeding and molecular field effects have been proposed as mechanisms of recurrence, evidence guiding surveillance strategies and therapeutic decision-making in dialysis-dependent patients or in those previously exposed to pelvic radiotherapy remains limited. This represents a clinically relevant gap in the literature, particularly in patients with minimal urinary output and altered symptom patterns.

Furthermore, this report highlights the value of multidisciplinary evaluation in complex uro-oncologic scenarios, where surgical radicality must be balanced against prior treatment morbidity, hemorrhagic risk, and overall prognosis.

As a single-case report, these observations are inherently limited and cannot be generalized. Nevertheless, documenting such sequential multifocal recurrence contributes to the growing body of evidence and may inform future research aimed at refining management strategies in high-risk populations.

## Conclusions

Recurrent multifocal urothelial carcinoma may necessitate extensive and sequential surgical interventions, particularly in patients with advanced chronic kidney disease. Early consideration of suspected hereditary cancer syndromes, such as Lynch syndrome, is essential in patients with multiple or early-onset urothelial malignancies, while recognizing that molecular confirmation is required before establishing a definitive diagnosis. A multidisciplinary approach is crucial to optimize oncological outcomes and preserve quality of life.
